# Protein kinase Cε: an oncogene and emerging tumor biomarker

**DOI:** 10.1186/1476-4598-8-9

**Published:** 2009-02-19

**Authors:** Michael A Gorin, Quintin Pan

**Affiliations:** 1 University of Miami, Miller School of Medicine, Miami, FL 33136, USA; 2 Department of Otolaryngology-Head and Neck Surgery, the Ohio State University Medical Center, Columbus, OH 43210, USA; 3 Arthur G James Cancer Hospital and Richard J Solove Research Institute, the Ohio State University Comprehensive Cancer Center, Columbus, OH 43210, USA

## Abstract

Members of the protein kinase C (PKC) family have long been studied for their contributions to oncogenesis. Among the ten different isoforms of this family of serine/threonine kinases, protein kinase Cε (PKCε) is one of the best understood for its role as a transforming oncogene. *In vitro*, overexpression of PKCε has been demonstrated to increase proliferation, motility, and invasion of fibroblasts or immortalized epithelial cells. In addition, xenograft and transgenic animal models have clearly shown that overexpression of PKCε is tumorigenic resulting in metastatic disease. Perhaps most important in implicating the epsilon isoform in oncogenesis, PKCε has been found to be overexpressed in tumor-derived cell lines and histopathological tumor specimens from various organ sites. Combined, this body of work provides substantial evidence implicating PKCε as a transforming oncogene that plays a crucial role in establishing an aggressive metastatic phenotype. Reviewed here is the literature that has led to the current understanding of PKCε as an oncogene. Moreover, this review focuses on the PKCε-mediated signaling network for cell motility and explores the interaction of PKCε with three major PKCε signaling nodes: RhoA/C, Stat3 and Akt. Lastly, the emerging role of PKCε as a tumor biomarker is discussed.

## Introduction to the protein kinase C (PKC) family

Several decades of research have documented that members of the protein kinase C (PKC) family of serine/threonine kinases are key signaling molecules involved in diverse cellular functions. Comprised of ten different isoforms, the PKC family is divided into three groups according to structural features and activation requirements. The classical isoforms (α, βI, βII, and γ) have an intact C1 diacylglycerol/phorbol ester binding domain and C2 calcium binding domain and thus require phospholipids and calcium for activation. The second group contains the novel isoforms (δ, ε, θ, and η,), which do not require calcium for their activation. The third group contains the atypical isoforms (ζ, and ι/λ), which can be activated in the absence of diacyglycerol and calcium.

Some of the many cellular processes regulated by PKCs include apoptosis, proliferation, migration, motility, chemo-resistance and differentiation (reviewed in [1-4]). Most notably, PKCs have been extensively studied in the context of oncogenesis. This body of literature first arose from work done in the 1980s when PKC was identified as an intracellular receptor for the tumor promoting phorbol esters [5-7]. Since these early reports, the discrete roles of many PKC isoforms in oncogenesis have been studied. It is now appreciated that each of the ten PKC isoforms is unique in its contribution to cancer development and progression. For example, within the context of an animal model of skin cancer, overexpression of PKCα has no effect on tumorigenesis [8], while the delta isoform suppresses tumor development [8,9]. In contrast, the epsilon isoform is severely oncogenic and promotes metastatic squamous cell carcinoma [10]. In other model systems, the varying roles of PKC isoforms have also been documented. PKCα has been described as an essential pro-proliferative and survival molecule in glioma cell lines [11]. Recently, PKCα was reported as a mediator of cell proliferation in head and neck cancer cell lines and as a predictive biomarker for disease-free survival in head and neck cancer patients [12]. Similarly, the iota isoform is now appreciated for its role in promoting cell motility/invasion [13] and proliferation [14].

Among the many PKC family members, several isoforms have been found to have paradoxical and tissue specific roles in tumor initiation. Of note, the contribution of PKCδ to oncogenesis has been contested through conflicting experimental findings. For example, several lines of evidence support PKCδ as an anti-proliferative molecule. These data include the work done in animal models of skin cancer [9] as well as the finding that PKCδ expression can revert transformed rat colonic epithelial cells towards a wild-type phenotype [15]. In contrast to this body of work, others have found that PKCδ promotes the survival of breast and lung cancer cells [16,17]. This discrepancy speaks to the complexity of understanding the roles of PKC family members in the context of oncogenesis. From the abundant literature on PKCs, it is clear that the function of PKC isoforms in oncogenesis is highly tissue dependent and thus it is critical to understand the role of each PKC isoform in the proper cellular context.

## PKCε as a transforming oncogene

In one of the first experimental works aimed specifically at exploring the oncogenic potential of PKCε, Mischak and colleagues overexpressed PKCε in NIH 3T3 fibroblasts [18]. These authors found that upon PKCε overexpression, cells grew at a faster rate and with increased densities and confluence. Cells transfected with PKCε also demonstrated an increased ability to grow in soft agar in the absence of phorbol 12-myristate 13-acetate (PMA). *In vivo*, 100% of mice injected with PKCε overexpressing NIH 3T3 cells developed tumors. Mice injected with PKCδ-expressing or untransfected NIH 3T3 cells did not develop tumors. Combined, these findings were among the first evidence demonstrating PKCε as an oncogene.

In the same year, Cacace and coworkers found that overexpression of PKCε in fibroblasts leads to an oncogenic phenotype *in vitro *and *in vivo *[19]. Similar to these earlier findings, Perletti *et al*. found that overexpression PKCε in FRC/TEX CL D (D/WT) colonic epithelial cells led to a metastatic phenotype which included morphological changes, increased anchorage independent growth and marked tumorigenesis in a xenograft model [20]. These reports set the stage for our current understanding of PKCε as a transforming oncogene.

## Proliferation and cell survival

Many of the details about the pro-tumorigenic signaling pathways modulated by PKCε have been elucidated. For example, PKCε is known to exert its oncogenic effects through modulation of the Ras signaling cascade [21-24], one of the best characterized signaling pathways in all of cancer biology (reviewed in [25]). Among the many downstream effects of Ras signaling includes increased cell cycling via up-regulation of cyclin D1 [26-29]. PKCε expression has been linked to activation of the cyclin D1 promoter and increased growth rates [30,31]

In addition to its influence on Ras signaling, PKCε has also been implicated in anti-apoptotic signaling pathways through the modulation of caspases and Bcl-2 family members [32-38]. Similarly, PKCε exerts its pro-survival effects by activating Akt/PKB [39-41]. Like the Ras signaling pathway, the pro-proliferative effects of Akt signaling are well described in the context of oncogenesis (reviewed in [42]). The role of PKCε in proliferation and survival signaling pathways has been extensively reviewed by Basu and Sivaprasad [43].

## Motility and invasion

In addition to promotion of proliferation and escape from apoptosis, dysregulation of cellular adhesion and motility are key components of a metastatic phenotype. The ability of a cell to migrate is essential for invasion of surrounding tissues as well as spread to distal sites. It is now appreciated that overexpression of PKCε leads to a highly motile and invasive phenotype. For example, Tachado and coworkers reported that expression of PKCε promotes a polarized cell morphology and *in vitro *cell invasion [44]. Moreover, these authors found that mice inoculated with NIH 3T3 cells overexpressing PKCε experienced tumor invasion of nearby tissues as well as liver metastases. Consistent with these findings, our group demonstrated that knockdown of PKCε with RNAi decreases *in vitro *invasion and motility [45,46], as well as incidence of lung metastases in a pre-clinical animal model of breast cancer [45].

Specific details of how PKCε modulates cell motility are beginning to emerge. It is now known that PKCε contains an actin binding domain [47]. Motile cells overexpressing PKCε have been observed to extend lamepidia-like protrusions [44]. The ability of PKCε to promote F-actin assembly in a cell-free system is compelling evidence for the role of PKCε in modulating outgrowth through actin polymerization [48]. PKCε has been observed to translocate to the cell membrane during the formation of focal adhesions [49]. This initial process of cellular adhesion to the extracellular matrix is known as cell spreading. During spreading, a cell undergoes a redistribution of adhesion and structural molecules. Once a cell has spread it may then take part in contact dependent functions such as motility. The expression of β1-integrin cytoplasmic domain connected to a non-signaling transmembrane domain inhibits the process of cell spreading [50]. PKCε is sufficient to rescue cell spreading in cells expressing non-signaling β1-integrin molecules [50]. This finding suggests PKCε signaling downstream of β1-integrin engagement with the extracellular matrix.

Ivaska *et al*. reported that PKCε plays a role in the return of endocytosed β1-integrin molecules to the plasma membrane [51]. The inhibition of PKCε with bisindolylmaleimide I (BIM-I) leads to an accumulation of intercellular β1-integrin/PKCε containing vesicles. Without the recycling of integrins, a necessary process for the advancement of cell's leading edge, a decrease in motility was observed. It is now known that phosphorylation of the cytoskeletal protein, vimentin, by PKCε is needed for the regulated release of integrin from recycled vesicles back to the plasma membrane [52].

In glioma cells, PKCε has also been found to be an important promoter of cell migration [53,54]. In adhesion assays, PKCε overexpressing cells were shown to adhere in greater numbers to laminin, vitronectin and fibronectin as compared to control cells [54]. PKCε expression was also responsible for lamellipodia formation. Similar to other reports, these authors found that PKCε interacts with β-integrin chains. In addition, it was found that RACK1, a scaffold protein associated with PKCε, is also a requirement of cell motility and forms a complex along with PKCε and β-integrin. This same group also found that extracellular-signal related kinase (ERK) localizes to sites of focal adhesions [53]. In PKCε overexpressing clones, an increase in the amount of activated phospho-ERK, a downstream molecule in the Ras signaling cascade, was observed. Cells transfected with an anti-sense PKCε cDNA showed the opposite pattern. Moreover, upon inhibition of the ERK signaling pathway, cells were less adhesive and motile in response to PMA stimulation.

**Figure 1:**
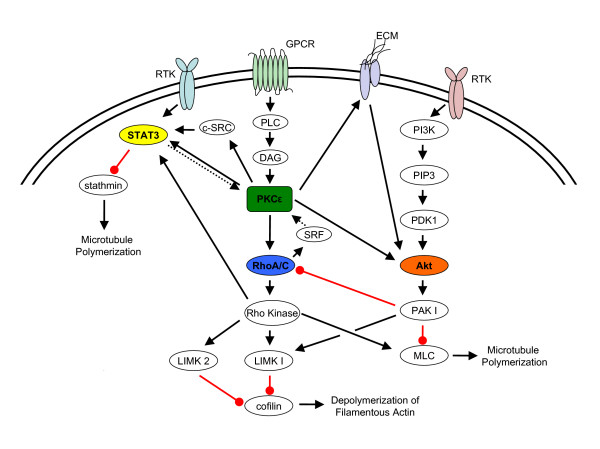
**Schematic model illustrating the PKCε signaling network for cell motility**. Arrow Black lines represent activation and Circle red lines represent inactivation. Arrow dashed lines represent putative interactions based on *in silico *transcription factor binding prediction software. All interactions are based on published literature as described in the text.

Our laboratory reported that PKCε-driven cell motility is mediated at least in part due to downstream activation of small Rho GTPases, specifically RhoA and/or RhoC [45,46]. RNAi-mediated knockdown of PKCε in head and neck cancer cells with elevated endogenous PKCε levels is sufficient to significantly impair cell motility [46]. Moreover, reconstitution of constitutive active RhoA or RhoC in these PKCε-deficient head and neck cancer cells rescued the loss-of-function motility defect providing direct evidence that RhoA and RhoC is downstream of the PKCε signaling cascade [46]. Our work, although novel, merely adds to the published literature on the linear signaling pathways mediated by PKCε to modulate cell motility. It is clear that signal transduction pathways must be approached through a systems biology perspective to capture the conditions necessary to drive a particular cellular event. To this end, we constructed a schematic model of the PKCε signaling network for cell motility using published results from our laboratory and other laboratories (Figure 1). This model contains three highly interconnected PKCε signaling nodes, Akt, RhoA/RhoC, and Stat3, with multiple positive and negative points.

Akt is activated through two phosphorylation events. PDK1 phosphorylates Akt at threonine 308 and recently, PKCε was identified as the kinase that phosphorylates Akt at serine 473 leading to full Akt activation [55]. There is evidence that Akt phosphorylates and activates PAK1 to enhance cell migration [56,57]. Interestingly, PAK1 was shown to inhibit the activity of NET1, a RhoA-specific guanine nucleotide exchange factor, suggesting that PAK1 will inhibit RhoA activation under certain conditions [58]. PAK1 phosphorylates myosin light chain kinase (MLCK) resulting in decreased MLCK activity and myosin light chain (MLC) phosphorylation in epithelial cells [59]. MLC is a component of myosin and involved in the assembly of actomyosin filaments for contraction. MLC is phosphorylated at Ser19 by myosin light chain kinase (MLCK) and Rho-kinase, a downstream effecter of RhoA and RhoC, to enhance myosin ATPase activity and muscle contraction [60,61]. PAK1 and Rho-kinase also directly regulates the activation of LIM domain kinases (LIMKs), a family of two serine/threonine kinases involved in actin cytoskeleton regulation. PAK1 phosphorylates LIMK1 at Thr508 and Rho-kinase, a downstream effector of RhoA and RhoC, phosphorylates LIMK2 at Thr505 resulting in an increase in LIMK1 and LIMK2 activity, respectively [62,63]. Activated LIMK1/LIMK2 inhibits the actin depolymerizing activity of cofilin by phosphorylation at the Ser3 residue of cofilin resulting in formation of stress fibers and focal adhesions for cell migration [63-65].

Recent reports demonstrated that PKCε directly phosphorylates Stat3 at Ser727 resulting in an increase in nuclear translocation and transcriptional activation of Stat3 [66-68]. Additionally, RhoA activation, mediated at least in part through Rho-kinase, results in an increase in Stat3 phosphorylation at Tyr705 and Ser727 and nuclear translocation [69]. Interestingly, Stat3 was reported to directly bind to the tubulin-binding region of stathmin to attenuate binding of stathmin to tubulin resulting in microtubule stabilization [70]. Stathmin is a ubiquitous cytosolic phosphoprotein involved in the regulation of the microtubule filament system by promoting tubulin filament depolymerization. Stathmin was reported to be phosphorylated at four serine residue, Ser16, Ser25, Ser38, and Ser63 by various kinases, including cyclin-dependent kinase, MAP kinase, and PKA [71,72]. Phosphorylation of stathmin at Ser16 and Ser63 was found to be necessary to optimally prevent stathmin from binding to tubulin resulting in microtubule stabilization [73].

*In silico *analysis using the Genomatix-MatInspector software identified Stat3 and serum response factor (SRF) transcription factor binding sites in the predicted PKCε promoter region. Stat3 nuclear translocation and transcriptional activity are regulated directly by PKCε and Rho-kinase [68]. In addition, SRF-mediated transcription is activated in response to RhoA and RhoC activation [74,75]. These observations is attractive from a molecular mechanism point of view as it suggests that a feed-forward mechanism, through PKCε-mediated increases in Stat3 and SRF transcriptional activities, may result in a sustained activation of the PKCε signaling network. Moreover, it is not known at this time, the contribution and requirement of each PKCε signaling module for promoting a motile cell phenotype. It is possible that there is redundancy in this system and that perhaps, only select nodes are indispensable to drive PKCε-mediated cell motility in cancer cells.

## Overexpression in various cancer types

The role PKCε in specific cancer types has been explored in great detail. To date, the overexpression of PKCε has been observed in a large number of cancer types (Table 1). The etiology of PKCε overexpression, however, remains elusive. To the best of our knowledge, no publications exist which document mutations responsible for PKCε overexpression. One group, however, has found 2p21 to be amplified in the rearranged DNA of WRO thyroid carcinoma cell lines and thyroid tumor samples [76,77]. This region on the short arm of chromosome 2 is home to the PKCε gene. The clinical value of this knowledge, however, is yet to be determined. Regardless of the origin of PKCε overexpression, it is clear that this protein is emerging through the literature as an important biomarker and potential drug target for many cancer types.

**Table 1:** PKCε overexpression in histopathological specimens

**Organ**	**Reference**
Bladder	84
Brain	87
Breast	45
Head and Neck	78
Lung	80
Prostate	67, 82

Our laboratory was the first to report that PKCε plays a causative role in establishing an aggressive, invasive, and motile phenotype in breast cancer [45]. Inhibition of PKCε by RNAi in MDA-MB231 cells, a highly metastatic breast cancer cell line with elevated PKCε levels, was sufficient to dramatically decrease cell proliferation, anchorage-independent colony formation, invasion, and motility *in vitro*. Similarly, MDA-MB231 cells transfected with RNAi against PKCε showed decreased tumor growth kinetics and a reduced incidence in the number of lung metastases in an orthotopic model of breast cancer. Moreover, using high-density tissue microarrays with tumor specimens from invasive ductal breast carcinoma patients, elevated PKCε was associated with high histologic grade, positive Her2/neu receptor status, and negative estrogen and progesterone receptor status. In addition, elevated PKCε was demonstrated to be a prognostic biomarker of poorer overall and disease-free survival.

PKCε has also been implicated in head and neck squamous cell carcinoma (HNSCC). In a small prospective study of 29 patients with primary squamous cell carcinomas of the oral cavity, elevated PKCε levels were significantly associated with disease relapse and decreased overall survival [78]. The authors of this study concluded that elevated PKCε may serve as a prognostic marker of aggressive disease in oral cancers. Consistent with this work, our laboratory reported that PKCε is elevated in a panel of HNSCC cell lines [46]. *In vitro*, HNSCC cell lines with high endogenous PKCε levels are associated with a highly invasive and motile phenotype. Additionally, RNAi-mediated silencing of PKCε in HNSCC with high endogenous PKCε levels resulted in a marked reduction in cell invasion and motility.

Elevated levels of PKCε have been documented in lung cancers. In the small cell lung carcinoma cell line (SCLC) NCI-N417, a constitutively active catalytic fragment of PKCε has been reported [79]. In non-SCLC cell lines (NSCLC), PKCε is associated with a chemo-resistant phenotype [32]. Aberrant PKCε levels have also been observed in histopathological lung tumor specimens [80]. Bae and colleagues have found PKCε to be overexpressed in >90% of NSCLC histological sections as compared to normal controls.

Like our group's work in both breast and head and neck cancer, inhibition of PKCε in NSCLC cells leads to decreased aggressive phenotype *in vitro *[80]. More specifically, cells treated with a dominant negative version of PKCε showed marked decreases in cell proliferation and anchorage-independent growth. Treatment of NSCLC cells with this dominant negative, or a RNAi against PKCε, resulted in a decrease in G_1_-S cell cycle transition. The authors attribute this arrest to an induction of p21/Cip1, a cyclin-dependent kinase inhibitor. From these results the authors point to PKCε as promoting dysregulation of the cell cycle in NSCLC.

In an animal model of prostate cancer, overexpression of PKCε in androgen-dependent LNCaP cells resulted in a transformation to an androgen-independent phenotype [81]. In both wild-type and castrated male nude mice, these cells undergo aggressive tumor growth. *In vitro*, PKCε overexpression conferred an increase in proliferation as well as resistance to apoptosis. Elevated PKCε levels have also been documented in clinical prostate cancer samples. One investigation found PKCε to be overexpressed in specimens of early prostatic adenocarcinomas obtained at time of radical prostatectomy [82]. As determined by high-density tissue microarrays, other investigators found increasing PKCε levels to correlate with tumor grade [67]. PKCε has also been found to be overexpressed in samples from other tissues of the urogenital tract. High PKCε levels have been detected in renal carcinoma cells [83] and in tumor specimens from the urinary bladder [84]. In the latter, increasing PKCε cells correlated with tumor grade [84].

PKCε has been shown to be an important player in driving squamous cell carcinoma (SCC) of the skin. Transgenic expression of PKCε under the control of the human keratin 14 promoter/enhancer has been shown to result in an 18-fold increase in PKCε [11]. This overexpression of PKCε drove the development of SSC in these animals with 40% of transgenic mice developing SSC of the skin as compared to 7% of controls. Histopathology revealed malignant invasion of tumors as well as metastases to regional lymph nodes. Overexpression of PKCε in FVB/N mice has also been demonstrated to predispose mice to SCC after ultra-violate radiation (UVR) exposure [85].

PKCε has been studied in malignancies of the central nervous system. In glioblastoma cell cultures, PKCε was found to be elevated between three to thirty times that of normal protein levels [86]. Consistent with this observation, Sharif and Sharif have reported that PKCε is overexpressed in a large number of astroglial cell lines [87]. Similarly, these authors report that PKCε is overexpressed in histological samples from anaplastic astrocytoma, globlastoma multiforme and gliosarcoma tumor samples. Interestingly, PKCε was not found in polocytic astrocytomas samples, a grade I malignancy.

The role or PKCε in myelo- and lympho- proliferative diseases has not been well studied. In one animal model, overexpression of PKCε in the epidermis of mice results in a myeloproliferative disease characterized by gross increases in the number eiosiniphils and neutrophils [88]. These mice show marked increases in serum levels of interleukin-5, interleukin-6 and G-CSF as well as infiltration of the lungs, liver and kidneys by the proliferative myeloid cells. The authors postulate that the overexpression of PKCε in these mice leads to an induction of cytokines which in turn promotes disease. Although this model system may prove to be important in studying myeloproliferative disease, the clinical significance of PKCε in this setting is still uncertain. At present, there is no existing literature to document PKCε overexpression in samples from patients with hematopoetic cancers. As such, the role of PKCε in such diseases of the hematopoetic compartment is unclear.

## Conclusion

Dysregulation of PKCε is a frequent genetic event associated with oncogenesis from multiple organ sites, including breast, prostate, and head and neck. PKCε is elevated in cell lines derived from patients and primary tumor specimens in numerous cancers providing substantial evidence for PKCε as a general transforming oncogene. Evidence to support PKCε as a prognostic biomarker for disease-recurrence and overall survival is beginning to emerge. Additional work is needed before the utility of PKCε as a value-added tool in the clinical decision-making process can be applied in the clinic. There is accumulating literature on the PKCε signaling pathway involved for cell invasion, motility, proliferation and survival; however, a system biology approach will be needed to begin to understand the conditions necessary to drive PKCε-mediated oncogenesis. Detailed understanding of the global PKCε signaling network, including temporal and intensity dynamics, is essential to identify "druggable" points in the signaling cascade to optimize therapeutic efficacy and minimize unwanted adverse effects. This approach will lead to the development of novel molecularly-targeted anti-cancer therapeutics which hopefully will improve the prognosis or quality of life for patients with aggressive metastatic disease.

## Competing interests

The authors declare that they have no competing interests.

## Authors' contributions

MAG and QP drafted the manuscript. All authors read and approved the final manuscript.
